# The bile acid-sensive ion channel (BASIC) is expressed in pancreatic α-cells and involved in glucagon secretion

**DOI:** 10.1007/s00424-026-03199-4

**Published:** 2026-07-29

**Authors:** Jingyu Yang, Stefan Gründer, Dominik Wiemuth

**Affiliations:** Institute of Physiology, RWTH Aachen University, D-52074 Aachen, Germany

**Keywords:** Bile acid-sensitive ion channel (BASIC), DEG/ENaC, Pancreatic α-cell, Glucagon

## Abstract

Pancreatic α-cells play a key role in glucose homeostasis through glucagon secretion. Here, we discover that the bile acid-sensitive ion channel (BASIC), a Na^+^ channel of the DEG/ENaC family, has a hitherto unrecognized role in α-cell function. We found that BASIC is mainly expressed in glucagon-positive α-cells of mouse islets. Genetic ablation of BASIC in mice reduced α-cell number, glucagon expression, and glucagon serum levels. These dramatic changes were accompanied by disturbed expression of α-cell specific transcription factors. Our findings identify BASIC as a previously unknown regulator of α-cell physiology.

**Supplementary Information:**

The online version contains supplementary material available at 10.1007/s00424-026-03199-4.

## Introduction

Pancreatic α-cells play a pivotal role in glucose homeostasis by secreting glucagon in response to hypoglycemia [26]. Dysregulation of α-cell mass or function contributes to the pathogenesis of metabolic diseases, including diabetes mellitus [13, 26]. While the molecular mechanisms governing β-cell identity and insulin secretion have been extensively studied [11, 15], these mechanisms are less well understood for α-cells and glucagon secretion [35].

Emerging evidence suggests that α-cells possess remarkable plasticity in response to metabolic stress. Under conditions of β-cell loss, α-cells can transdifferentiate into insulin-producing β-like cells, contributing to functional compensation. Conversely, chronic hyperglycemia and inflammatory stress can induce α-cell dysfunction, including impaired glucagon secretion and altered transcriptional programs [5].

Preservation of α-cell identity and survival is critical for maintaining islet architecture and systemic glucose regulation. However, the key regulators of α-cell stability are largely unknown [4, 12, 25]. In particular, how intrinsic factors coordinate α-cell survival, transcriptional programming, and secretory function under physiological and pathological conditions remains to be elucidated [14, 21].

The bile acid-sensitive ion channel (BASIC) is a member of the DEG/ENaC family, most closely related to acid-sensing ion channels (ASICs) [23, 30, 40]. BASIC is a Na⁺-selective, amiloride-sensitive channel that is strongly inhibited by extracellular Ca²⁺ (IC₅₀ ≈ 10 µM for rBASIC). Its open probability is low under physiological conditions [39, 40]. In contrast to ENaC, which is constitutively active, and ASICs, which are proton-gated, BASIC lacks a known endogenous ligand. BASIC activity can be unmasked by Ca²⁺ removal or gain-of-function mutations, revealing robust Na⁺-selective currents [10, 31, 32]. Although its physiological agonist remains unclear, bile acids have been identified as potent activators [16, 36, 38], likely acting indirectly by altering plasma membrane properties, and fenamates can serve as artificial agonists [37]. Pharmacologically, BASIC is blocked by diarylamidines and nafamostat at low micromolar concentrations [22, 31, 37]. The channel is predominantly expressed in liver, intestine, and bile duct epithelium [30, 38], where it plays a potential role in epithelial Na⁺ transport [36]. Nevertheless, the precise physiological function of BASIC remains to be elucidated.

Expression of BASIC in the endocrine pancreas has not yet been described. Here, we demonstrate that, within the pancreas, BASIC is mainly expressed in α-cells and investigate its role in their biology. We show that deletion of BASIC alters islet morphology, and reduces glucagon secretion and α-cell number. Moreover, we find that loss of BASIC is accompanied by transcriptional changes in α-cell marker genes.

Taken together, our findings uncover a previously unrecognized role for BASIC in α-cell function and islets homeostasis.

## Results

### BASIC is mainly expressed in α-cells within pancreatic islets

**Fig. 1:**
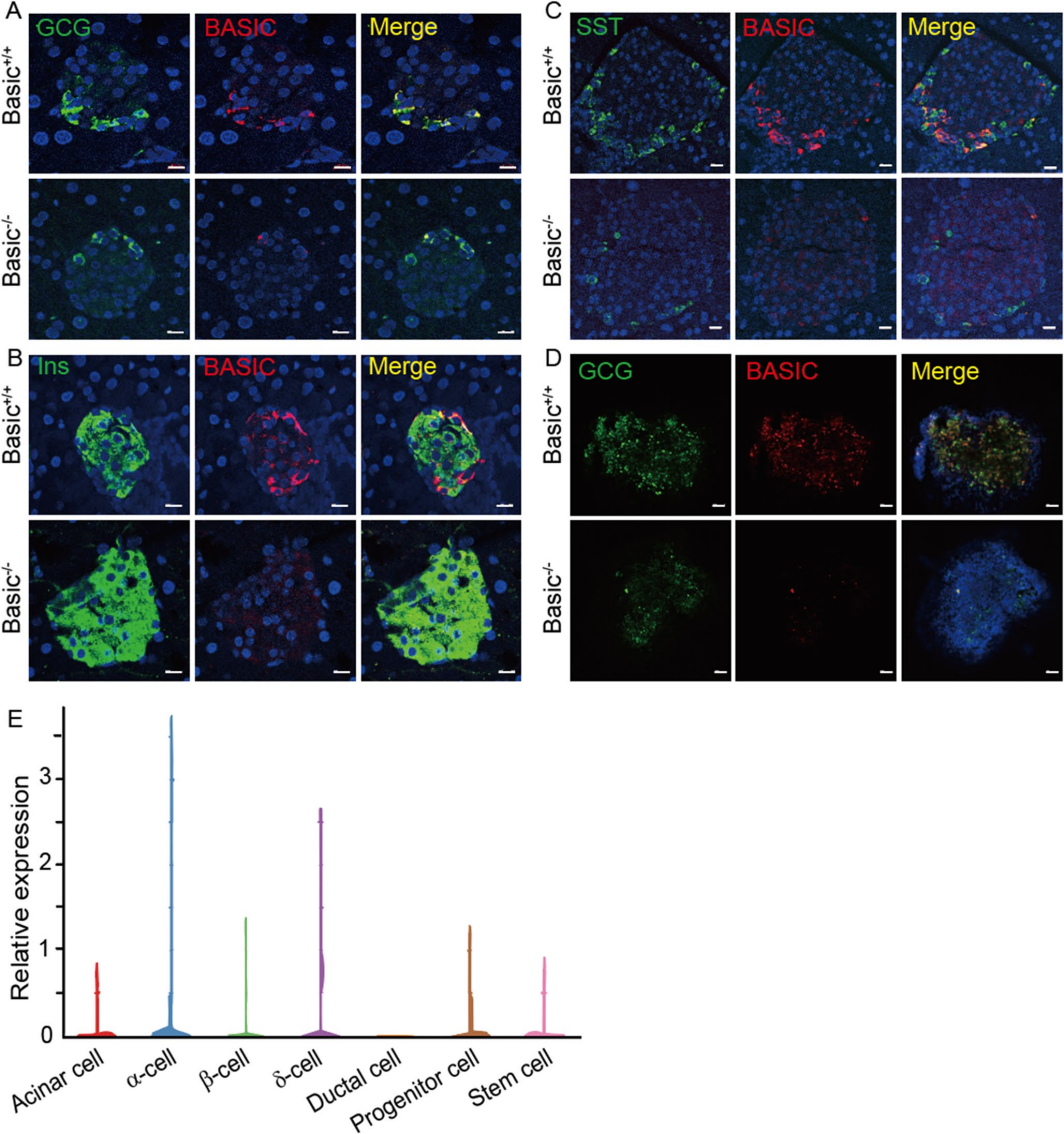
BASIC is mainly expressed in α-cells of pancreatic islets. (**A-C**) Representative confocal images of paraffin-embedded pancreatic sections from *Basic*^⁺/⁺^ and *Basic*^⁻/⁻^ mice co-stained for (**A**) glucagon (GCG, green) and BASIC (red), (**B**) insulin (Ins, green) and BASIC (red), and (**C**) somatostatin (SST, green) and BASIC (red). Nuclei were counterstained with DAPI (blue). (**D**) Representative confocal images of whole-mount stainings of isolated islets showing co-localization of BASIC and GCG in islets from *Basic*^⁺/⁺^ and *Basic*^⁻/⁻^ mice. Scale bars, 20 μm. (**E)**. Single-cell RNA-sequencing analysis of human pancreatic cell populations shows that BASIC expression is predominantly enriched in α-cells, with lower levels detected in β-cells, δ-cells, and other pancreatic cell types. Data were visualized using the Cell-omics Data Coordinate Platform (CDCP; dataset SCDS0000366; sample GSM5979689), based on the original dataset from GEO: GSE199636

Immunofluorescence analysis of pancreas sections from mice using a polyclonal anti-BASIC antibody revealed that BASIC predominantly co-expressed with glucagon-positive (GCG)⁺ α-cells but was largely absent from somatostatin-positive (SST)⁺ δ-cells and insulin-positive (Ins)⁺ β-cells (Fig. 1A–C). BASIC immunoreactivity was not detected in islets from *Basic⁻/⁻* mice, whereas GCG, SST, and Ins were detectable in both genotypes (Fig. 1A–C). Staining of isolated islets confirmed the co-localization of BASIC and GCG in *Basic⁺/⁺* islets, while no BASIC signal was detected in *Basic⁻/⁻* islets (Fig. 1D). To further support the cellular distribution of BASIC expression within the pancreas, we analyzed publicly available single-cell RNA-sequencing data of human pancreas [17]. These data support α-cells as the predominant site of BASIC expression also in the human pancreas (Fig. 1E). These results uncover a hitherto unrecognized expression of BASIC in pancreatic islets, which is mainly found in α-cells.

### BASIC deficiency reduces α-cell number and impairs islet morphology

**Fig. 2:**
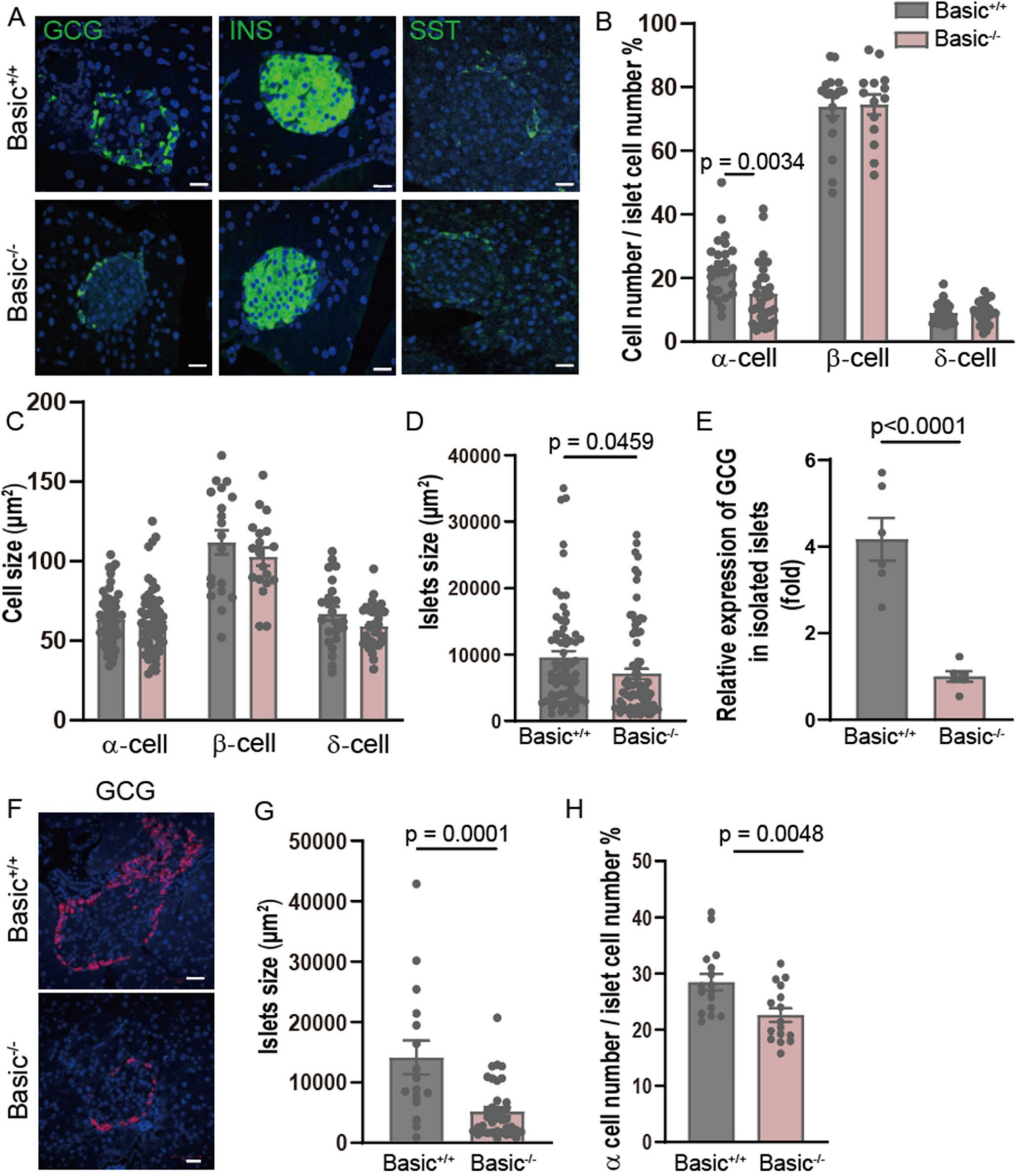
BASIC deficiency leads to a reduction in α-cell number and islet size. (**A**) Representative confocal images of paraffin-embedded pancreatic tissue sections stained for glucagon (GCG), insulin (INS), or somatostatin (SST) (green). Nuclei were counterstained with DAPI (blue). (**B**) Quantification of α-, β-, and δ-cell numbers as a percentage of total islet cells. (**C**) Quantification of the size of individual α-, β-, and δ-cells. (**D**) Quantification of total islet size. (**E**) RT-qPCR analysis of GCG mRNA expression in isolated islets. (**F**) Representative confocal images of GCG RNAscope staining of pancreatic islets. (**G**) Quantification of total islet size identified by GCG staining shown in (**F**). (**H**) Quantification of GCG⁺ cell number as a percentage of total islet cells from figure F. All analyses were performed in *Basic*^*⁺/⁺*^ and *Basic*^*⁻/⁻*^ mice. Scale bars, 20 μm. Data are presented as mean ± SEM; individual data points represent biological replicates. p-values were determined by unpaired two-tailed t-test

We noticed a reduced staining for GCG in *Basic*^−/−^ islets. To investigate this observation in more detail, we examined the abundance and morphology of endocrine cell types in *Basic*⁻/⁻ mice. Quantitative analysis of immunofluorescence experiments indeed revealed a significant decrease in GCG⁺ α-cells in *Basic*^⁻/⁻^ islets, whereas the populations of INS⁺ β-cells and SST⁺ δ-cells were largely preserved (Fig. 2A and B). Other cell types were not analyzed and may show alterations. Importantly, the size of individual α-, β-, and δ-cells was not different between *Basic*^+/+^ and *Basic*^⁻/⁻^ mice (Fig. 2C), suggesting that reduced GCG^+^ staining reflects α-cell loss rather than reduction in cell size.

Consistent with the decline in α-cell number, the total islet area was significantly reduced in *Basic⁻*^*/*^*⁻* mice compared to controls (Fig. 2D). Moreover, RT-qPCR analysis showed a substantial decrease in GCG mRNA levels in *Basic*^⁻/⁻^ islets (Fig. 2E). RNAscope staining for GCG further demonstrated a pronounced shrinkage of GCG⁺ areas and a reduction in α-cell numbers in *Basic*^⁻/⁻^ islets (Fig. 2F and H). Together, these findings indicate that loss of BASIC reduces α-cell number and affects islet architecture.

### In adult mice, BASIC loss changes the expression of key transcription factors in α-cells

Next, we investigated whether BASIC loss affects α-cell proliferation and assessed cellular proliferation markers and analyzed the expression of transcription factors critical for α-cell fate determination.

**Fig. 3:**
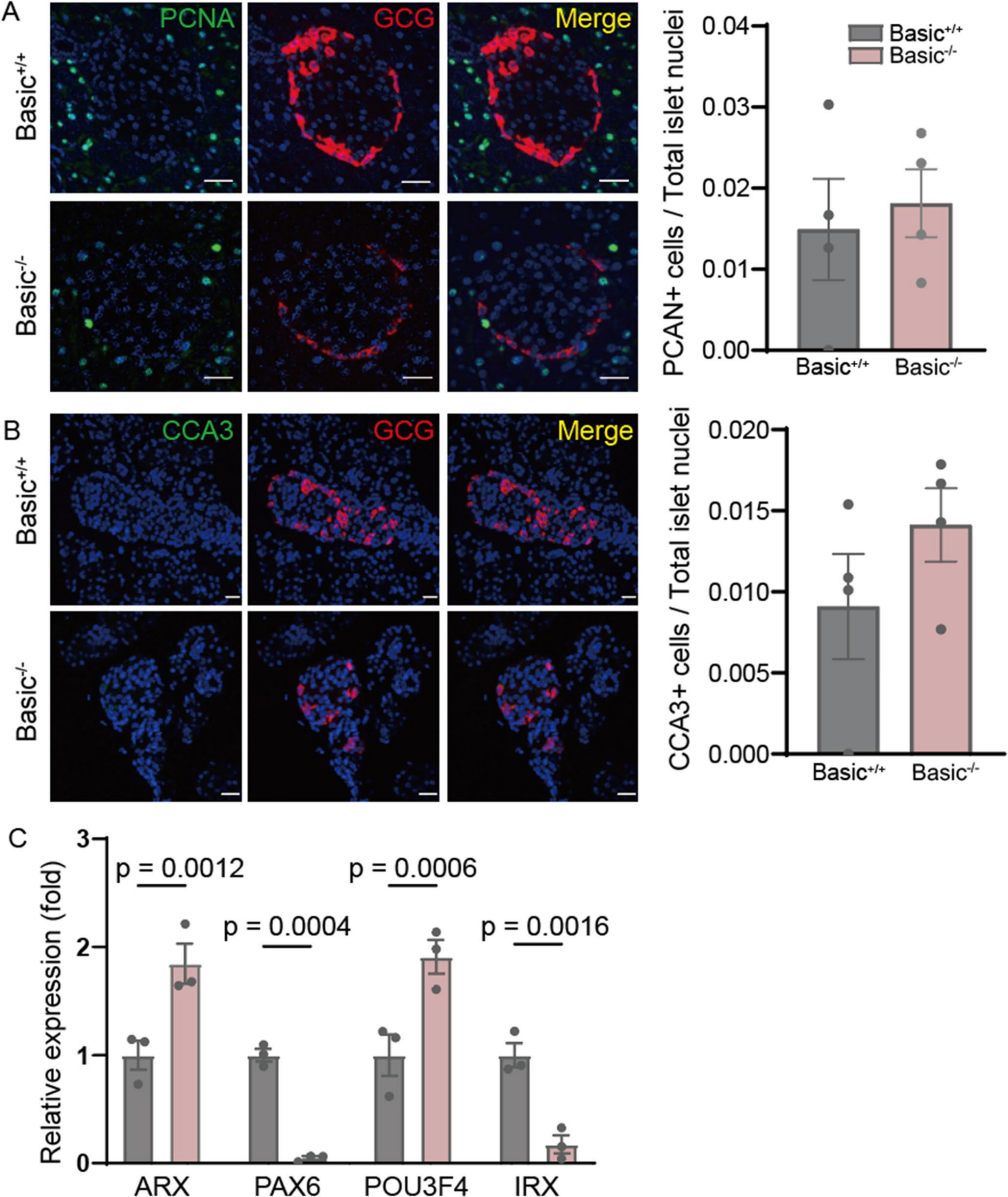
BASIC deficiency alters α-cell gene expression without affecting proliferation in adult mice. (**A–B**) Representative confocal images of pancreatic islets from paraffin-embedded pancreatic sections of adult *Basic*^⁺/⁺^ and *Basic*^⁻/⁻^ mice stained for (**A**) proliferating cell nuclear antigen (PCNA, green) and glucagon (GCG, red), or (**B**) cleaved-caspase 3 (CCA3, green) and GCG (red). Nuclei were counterstained with DAPI (blue). Scale bars, 20 μm. Corresponding quantification of PCNA⁺ (**A**) and CCA3⁺ (**B**) cells within islets are shown as a fraction of total islets cells. Each data point represents an individual mouse. (**C**) RT-qPCR analysis from isolated islets of transcription factors associated with α-cell identity (ARX, PAX6, POU3F4, and IRX) from adult *Basic*^⁺/⁺^ and *Basic*^⁻/⁻^ mice. Data are presented as mean ± SEM; individual data points represent biological replicates. p-values were determined by unpaired two-tailed t-test

Immunofluorescence staining of pancreatic islets from adult mice revealed comparable levels of the proliferating cell nuclear antigen (PCNA), an essential replication factor, and cleaved caspase-3 (CCA3), a classical marker for cell apoptosis, between *Basic*^⁺/⁺^ and *Basic*^⁻/⁻^ mice (Figs. 3A and B), indicating that the decrease in α-cell number is not due to altered proliferation or apoptosis at this stage.

We then examined the expression of key transcription factors associated with α-cell proliferation. Aristaless-related homeobox (ARX) and POU class 3 homeobox 4 (POU3F4) are central drivers of α-cell differentiation and identity, while paired box protein 6 (PAX6) regulates glucagon transcription and α-cell function, and Iroquois homeobox (IRX) factors contribute to α-cell fate determination and stabilization of lineage-specific gene expression. RT-qPCR analysis demonstrated that the mRNA levels of ARX and POU3F4 were significantly elevated, whereas PAX6 and IRX were markedly reduced in *Basic*^⁻/⁻^ islets compared to controls (Fig. 3C), suggesting that BASIC loss alters transcriptional programs at the islet level, including regulators of α-cell fate, which may reflect changes in α-cell number and potential defects in α-cell maintenance.

### In postnatal mice, BASIC deficiency promotes α-cell death and impairs islet development

Next, we examined whether the changes in α-cell number and key transcription factor expression observed in adult mice already occur at an early developmental stage. To this end, we analyzed pancreatic islets from postnatal day 7 (P7) mice.

**Fig. 4:**
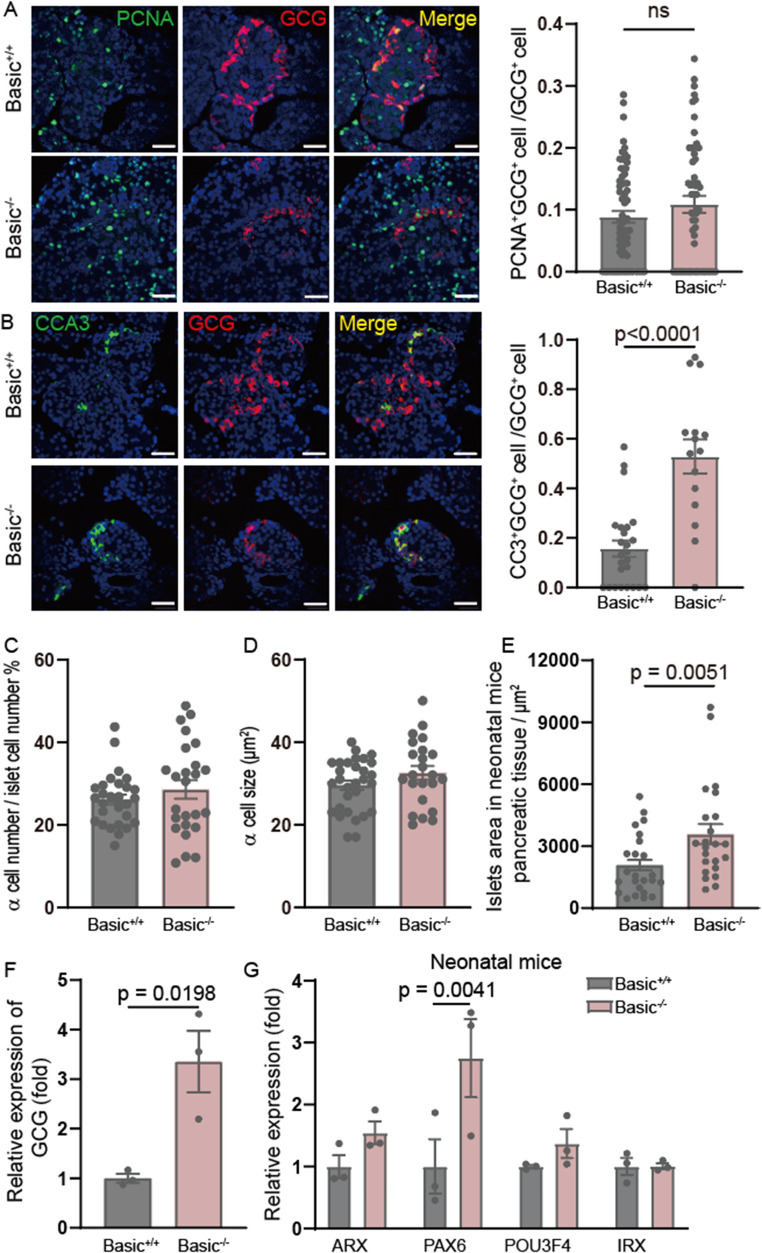
BASIC deficiency promotes α-cell death in neonatal mice. (**A, B**) Representative confocal images of pancreatic islets from paraffin-embedded pancreatic sections of P7 *Basic*^⁺/⁺^ and *Basic*^⁻/⁻^ mice stained for (**A**) proliferating cell nuclear antigen (PCNA, green) and glucagon (GCG, red), or (**B**) cleaved caspase-3 (CCA3, green) and GCG (red). Nuclei were counterstained with DAPI (blue). Scale bars, 20 μm. Corresponding quantification of PCNA⁺GCG⁺ (**A**) and CCA3⁺GCG⁺ (**B**) α-cells are shown as a fraction of total GCG⁺ cells. (**C**) Quantification of α-cell number as a percentage of total islet cells in *Basic*^⁺/⁺^ and *Basic*^−/−^ mice. (**D**) Quantification of individual α-cell size. (**E**) Quantification of total islet area per pancreatic tissue section (**F**) RT-qPCR analysis of GCG mRNA expression in pancreatic tissues from P7 mice. (**G**) RT-qPCR analysis of α-cell transcription factors (ARX, PAX6, POU3F4, IRX) in isolated islets from P7 mice. Data are presented as mean ± SEM; individual data points represent biological replicates. p-values were determined by unpaired two-tailed t-test

Immunofluorescence staining of pancreatic sections showed no significant difference in the proportion of PCNA⁺GCG^+^ cells between *Basic*^⁺/⁺^ and *Basic*^⁻/⁻^ mice (Figs. 4A). Furthermore α-cell number and size of individual α-cells were largely comparable (Figs. 4C-D). However, the overall islet area was markedly increased in P7 *Basic*^−/−^ mice (Fig. 4E).

Gene expression analysis revealed a selective upregulation of PAX6 in BASIC-deficient islets, which is in clear contrast to adult mice, while ARX, POU3F4, and IRX expression remained unchanged (Fig. 4G). Interestingly, GCG mRNA levels were significantly increased in *Basic*^⁻/⁻^ pancreatic tissues. (Fig. 4F).

Notably, staining of pancreatic sections for CCA3, a marker of apoptosis, demonstrated a substantial increase in CCA3⁺GCG⁺ α-cells in *Basic*^⁻/⁻^ mice (Figs. 4B), indicating that BASIC loss leads to α-cell apoptosis during early postnatal development. These findings suggest that BASIC is critical maintaining islet integrity during neonatal life.

### BASIC modulates α-cell glucagon secretion and proliferation

**Fig. 5:**
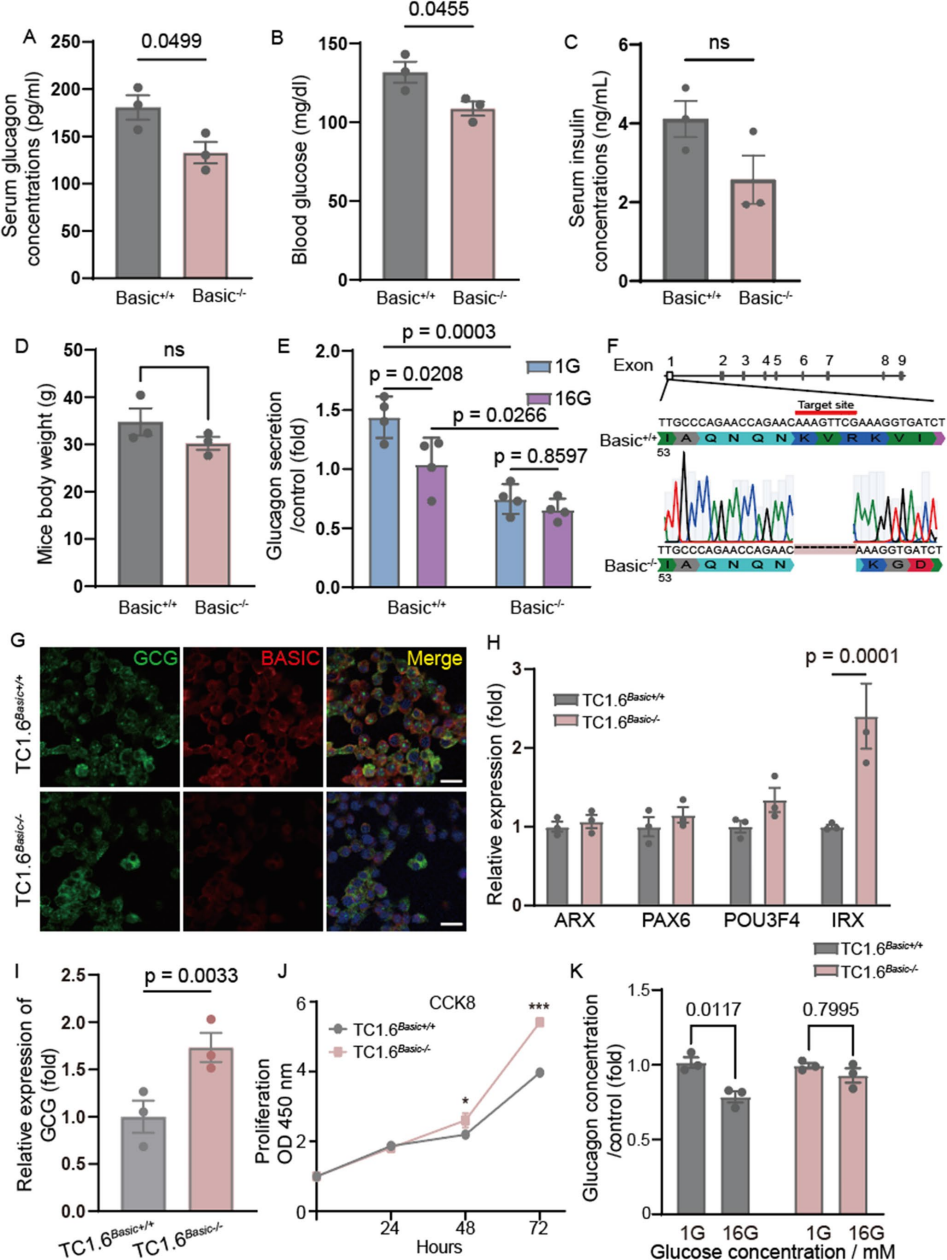
BASIC influences α-cell glucagon secretion and cellular function. (**A**) Serum glucagon concentrations. (**B**) Blood glucose levels measured immediately after sacrifice. (**C**) Serum insulin concentrations and (**D**) Body weight in adult *Basic*⁺^/^⁺ and *Basic*⁻^/^⁻ mice. (**E**) Glucagon secretion from isolated islets of *Basic⁺*^*/*^*⁺* and *Basic*⁻^/^⁻ adult mice at 1 mM (1G, low glucose) and 16 mM (16G, high glucose) glucose conditions. (**F**) Identification of targeted genome editing in TC1.6 cells induced by CRISPR/Cas9. Schematic representation of the exon–intron structure of the mouse BASIC gene and the Basic^+/+^ and Basic^−/−^ alleles. The 8 bp deletion induced a frameshift resulting in a premature stop codon at position 101. (**G**) Representative immunofluorescence images of TC1.6^*Basic+/+*^ and TC1.6^*Basic−/−*^ cells stained for GCG (green) and BASIC (red). Nuclei were counterstained with DAPI (blue). Scale bars, 20 μm. (**H**) RT-qPCR analysis of TC1.6^*Basic*+/+^ and TC1.6^*Basic*−/−^ cells for α-cell transcription factors (ARX, PAX6, POU3F4, IRX). (**I**) RT-qPCR analysis of GCG mRNA expression in TC1.6^*Basic*+/+^ and TC1.6^*Basic*−/−^ cells. (**J**) Proliferation assay (CCK8) showing proliferation of TC1.6^*Basic*+/+^ and TC1.6^*Basic*−/−^ cells over 72 h. (**K**) Glucose-regulated glucagon secretion in TC1.6^*Basic*+/+^ and TC1.6^*Basic*−/−^ cells under 1G and 16G conditions. Data are presented as mean ± SEM; individual points represent biological replicates. p-values determined by two-way ANOVA multiple comparisons test (**B**) and unpaired two-tailed t-test (**A, D-G**)

We next examined how BASIC affects α-cell function and glucagon secretion under both basal and stress conditions. Body weight did not differ significantly between Basic^+/+^ and Basic^−/−^ mice (Fig. 5D). In contrast, Basic^−/−^ mice showed a modest but significant reduction in blood glucose levels (Fig. 5B).

To assess whether this phenotype was associated with altered circulating islet hormones, serum insulin and glucagon levels were measured. Serum insulin showed a decreasing trend in Basic^−/−^ mice but did not reach statistical significance (Fig. 5C). By contrast, serum glucagon levels were significantly reduced in Basic^−/−^ mice (Fig. 5A). Similarly, isolated pancreatic islets from *Basic*^⁻/⁻^ mice exhibited impaired glucose-regulated glucagon secretion, particularly under low-glucose conditions (Fig. 5E) compared with *Basic*^+/+^ control.

We further explored the role of BASIC using TC1.6 cells, a mouse α-cell line expressing GCG. To directly assess the impact of genetic loss of BASIC on these cells, we generated a BASIC-deficient TC1.6 cell line using CRISPR-Cas9. Immunofluorescence confirmed successful deletion of BASIC while retaining GCG expression (Fig. 5F-G). Transcriptional analysis revealed that BASIC-deficiency upregulated IRX expression, with minimal changes in ARX, PAX6, and POU3F4 expression (Fig. 5H). Additionally, GCG mRNA levels were significantly elevated in *Basic*^−/−^ cells (Fig. 5I), similar to the situation in neonatal mice and opposite to adult mice. The estimated doubling time of TC1.6^Basic−/−^ cells was shorter, approximately 20 h, while that of TC1.6^Basic+/+^ cells was approximately 27 h, indicating that proliferation was accelerated after loss of BASIC (Fig. 5J).

High glucose concentrations decrease glucagon secretion in TC1.6 cells [24]. In agreement, *Basic*^+/+^ cells exhibited a decreased glucagon secretion at high-glucose conditions (16G) compared to low-glucose (1G). In contrast, *Basic*^−/−^ cells showed no significant difference in glucagon secretion between 1G and 16G (Fig. 5K), indicating impaired glucose-responsiveness.

Together, these findings suggest that BASIC is involved in the regulation of α-cell glucagon secretion and confirm that its absence leads to altered expression of key transcription factors of α-cells.

## Discussion

In this study, we report a previously unrecognized expression of BASIC in pancreatic α-cells and show that genetic deletion of BASIC leads to reduced α-cell numbers, impaired glucagon secretion, and alterations in the islet transcriptional program.

To determine which endocrine cell types express BASIC, we performed cell-based quantitative analyses on pancreatic sections co-stained for BASIC and cell markers (glucagon, insulin, or somatostatin). Because our objective was to define cell-type–specific expression rather than subcellular co-localization within individual cells, we quantified BASIC signal at the single-cell level by scoring its presence within marker-positive cells. We first demonstrated that in pancreatic islets, BASIC is mainly expressed in α-cells. While β- and δ-cell populations were unaffected, BASIC deficiency significantly reduced the proportion of α-cells, most likely reflecting gradual α-cell death. The unchanged size of individual α-cells further supports the notion that the reduction was due to cell loss rather than atrophy. Notably, the reduction in α-cell percentage was not associated with a compensatory increase in β- or δ-cells, arguing against endocrine cell fate conversion and supporting a selective impairment of α-cell maintenance.

Further analysis revealed that, in adult mice, BASIC deficiency led to a marked decrease in islet size, accompanied by a reduction in glucagon mRNA expression. In neonatal mice, BASIC loss resulted in increased α-cell apoptosis, as evidenced by elevated cleaved caspase-3 staining, without detectable changes in proliferation rates. These findings suggest that BASIC deficiency may impair α-cell survival during early postnatal development, a period when islet architecture is being established.

At the transcriptional level, the loss of BASIC was associated with divergent regulation of α-cell fate regulators, including upregulation of ARX and POU3F4 alongside downregulation of PAX6 and IRX. As these measurements were performed on whole islets, the observed changes must be interpreted in the context of altered endocrine cell composition. The reduced PAX6 expression may partly reflect decreased overall islet cellularity, whereas increased ARX and POU3F4 expression could represent context-dependent or compensatory transcriptional responses within the remaining α-cell population. Together, these findings indicate disruption of islet transcriptional programs linked to α-cell identity and homeostasis.

Functionally, BASIC deficiency is associated with modest alterations in glucose homeostasis. Body weight was not significantly different between Basic^+/+^ and Basic^−/−^ mice, indicating that the observed reduction in blood glucose is unlikely to be primarily driven by differences in overall growth or nutritional status. Basic^−/−^ mice showed lower circulating glucagon levels, whereas insulin levels showed only a non-significant downward trend, suggesting that the glucose phenotype may be more closely related to changes in the glucagon axis than to enhanced insulin action. This is consistent with the results of the histological analysis (Fig. 2). Interestingly, despite the reduction in α-cell number in BASIC-deficient mice, both neonatal islets and BASIC-deficient TC1.6 α-cell lines exhibited increased GCG transcription and enhanced proliferative capacity. These observations suggest that BASIC loss may lead to compensatory changes in α-cell transcription and proliferation, with the increased islet size in neonatal mice supporting a transient expansion of α-cell populations.

However, in vivo, this initial proliferative response may paradoxically promote cellular stress and trigger apoptosis, ultimately leading to a net loss of α-cells. This hypothesis is consistent with the observed increase in cleaved caspase-3 (CCA3) staining in BASIC-deficient neonatal islets, indicating elevated apoptosis. Such compensatory dynamics, wherein hyperproliferation predisposes cells to stress-induced death, have been reported in β-cells under developmental or pathological stress [8, 29, 41].

Therefore, these findings suggest that BASIC deficiency is associated with an imbalance in α-cell homeostasis, characterized by enhanced proliferative signals but insufficient survival capacity. Future studies will be needed to determine whether and how BASIC directly regulates pathways related to cell cycle control and stress responses.

While systemic neuroendocrine factors may contribute to these effects, the changes observed upon BASIC deletion appear largely α-cell-related. The effects are most evident in α-cell number and transcriptional regulators such as Pax6, Arx, IRX and Pou3f4, together with GCG expression, suggesting at least a partial cell-intrinsic component. During the early postnatal period, however, islet innervation and hormonal feedback circuits are still maturing, and α-cell secretion is not yet fully integrated with neuronal and paracrine inputs [3, 6, 33]. Such developmental immaturity may amplify the apparent impact of BASIC loss in newborn mice compared with adults. Future studies employing α-cell-specific GCG-Cre models or targeted denervation will be important to definitively distinguish BASIC in α-cell-autonomous from systemic contributions.

Increasing evidence shows that some ion channels perform signaling roles beyond ion transport, particularly in regulating gene expression [2, 9, 18]. For example, the potassium channel KCNQ1 modulates Wnt/β-catenin signaling by forming a membrane complex with β-catenin, retaining it at the plasma membrane and preventing its nuclear translocation [2, 27]. Likewise, the channel kinase TRPM7 controls the transcriptional activities of STAT3 and SOX9 through Ca²⁺-dependent signaling and direct phosphorylation of downstream regulators [9, 18]. Our finding that BASIC deficiency alters Pax6 expression suggests a potential link between BASIC-mediated membrane signaling and transcriptional regulation, possibly through modulation of the local ionic microenvironment or protein complex organization.

Although our findings demonstrate that BASIC influences α-cell number, gene expression, and glucagon secretion, the precise mechanism by which BASIC regulates α-cell function remains unknown. Ion channels play fundamental roles in islet cell physiology, particularly in regulating electrical activity, Ca²⁺ signaling, and hormone release [7, 20]. For example, ATP-sensitive K⁺ channels and voltage-gated Ca²⁺ channels are essential for glucose-stimulated insulin secretion in β-cells, while voltage-gated Na⁺ channels, and K⁺ channels modulate β-cell excitability and insulin granule exocytosis [28, 34]. Similarly, BASIC may influence α-cell activity through its ion conductance properties or by indirectly affecting membrane potential, cellular signaling, or cell-cell communication within the islet microenvironment.

α-cells avidly utilize glutamine and glutamate, co-secrete glutamate with glucagon, and increase glucagon secretion in response to glutamine [1]. Although glutamine did not directly modulate BASIC activity in our assays (data not shown), BASIC may nevertheless influence amino acid–evoked α-cell responses by tuning membrane excitability and stimulus–secretion coupling. Future studies should therefore investigate glutamine-stimulated glucagon secretion together with α-cell Ca²⁺ dynamics and intra-islet paracrine signaling in the BASIC knockout model to define the physiological contexts in which BASIC becomes functionally relevant.

Overall, our results position BASIC as a critical regulator of α-cell function and homeostasis. By preserving α-cell number, preventing inappropriate transcriptional reprogramming, supporting survival during development, and maintaining functional responsiveness, BASIC may affect the integrity of glucagon-producing cells and the maintenance of glucose homeostasis. However, the current data remain largely correlative, and direct causal relationships require further validation through glucose and insulin tolerance tests, hepatic gluconeogenic analyses, and lineage-tracing studies. Future studies investigating the downstream signaling pathways controlled by BASIC may reveal new strategies for modulating α-cell function in metabolic diseases such as diabetes.

## Methods

### Mouse model

Cryo-conserved sperm from the mouse line C57BL/6N-A^tm1Brd^ Asic5^tm1a(KOMP)Mbp^/MbpMmucd mice was purchased from the Mutant Mouse Resource & Research Centers (MMRRC). In-vitro fertilization was conducted by the Core Facility “Transgenic Facility” (TF) of the IZKF Aachen at the Medical Faculty of RWTH Aachen. Animal care was conducted following protocols approved by the State Office for Consumer Protection and Food Safety (LAVE) of North Rhine-Westphalia (NRW), Germany and was performed in accordance with LAVE NRW guidelines. Mice were housed in a conventional facility at 21 °C on a 12-hour light/12-hour dark cycle with unrestricted access to food and water. Genotyping of C57BL/6N-A^tm1Brd^ Asic5^tm1a(KOMP)Mbp^/MbpMmucd mice was conducted according to the MMRRC protocol for this strain. All mice used in this study were male.

### Islet isolation

Pancreatic islets were isolated from adult (8–12 weeks old) mice by collagenase (Liberase™) digestion followed by density gradient centrifugation and handpicking under a stereomicroscope. Islets were cultured overnight in RPMI-1640 medium supplemented with 10% fetal bovine serum (FBS) and 1% penicillin-streptomycin before use.

### Immunofluorescence staining

Pancreatic tissues were fixed in 4% paraformaldehyde at 4 °C overnight, dehydrated through graded ethanol series, cleared in xylene, and embedded in paraffin. Paraffin-embedded tissues were sectioned at 5 μm thickness using a microtome. Sections were deparaffinized in xylene, rehydrated through descending grades of ethanol to water, and subjected to antigen retrieval by Proteinase K at 37 °C for 10 min. After blocking with 5% bovine serum albumin (BSA) in phosphate-buffered saline (PBS) for 1 h at room temperature, sections were incubated with primary antibodies against glucagon (GCG, 1:100, A14609, Abclonal), insulin (INS, 1:100, A2090, Abclonal), somatostatin (SST, 1:100, A20617, Abclonal), BASIC (1:100, custom-made polyclonal antibody, epitope: ILETIQRTSPPQAV [38]), proliferating cell nuclear antigen (PCNA, 1; 500, 10205-2-AP, Proteintech), and cleaved caspase-3 (CCA3, 1:400, 9661 T, Cell Signaling Technology) overnight at 4 °C. After washing, sections were incubated with Alexa Fluor-conjugated secondary antibodies, and nuclei were counterstained with DAPI. Confocal images were acquired using a Zeiss LSM 710 laser scanning confocal microscope.

### RNAscope

RNAscope in situ hybridization was performed using ACD Biotechne (Oxford, UK) following the manufacturer’s protocol with minor modifications. Paraffin sections were deparaffinized, rehydrated, and subjected to target retrieval and protease treatment under optimized conditions for pancreas and liver. Target probes were hybridized, followed by standard Multiplex FL v2 amplification and fluorophore labeling. Slides were counterstained with DAPI, mounted with antifade medium, and stored at 2–8 °C until imaging.

### Quantitative analysis of cell number, size, and islet area

Cell number, size, and islet area were quantified using the software Zeiss Zen 3.9. α-, β-, and δ-cells were manually identified based on immunofluorescence markers assessing BASIC signal within hormone-positive cells to define cell-type–specific expression. Cell size was determined by measuring the cross-sectional area. Total islet area was defined as the DAPI-positive area encompassing endocrine clusters.

### Quantification of PCNA/GCG and CC3/GCG co-localization

Immunofluorescence images were analyzed in Fiji/ImageJ using identical acquisition and analysis parameters across all groups. Islets were identified based on DAPI-dense endocrine cell clusters and overall islet morphology, while GCG staining was used to identify α-cells within each islet. DAPI-positive nuclei within the islet boundary were used as cell identifiers. Background fluorescence was estimated from non-islet regions within the same section and subtracted consistently across images. The same thresholding criteria were applied to all images within the same staining batch.

GCG-positive α cells were defined as cells showing cytoplasmic GCG signal spatially associated with a DAPI-positive nucleus. PCNA-positive cells were identified by nuclear PCNA signal overlapping with DAPI-positive nuclei. CC3-positive cells were identified by CC3 staining signal above background that was spatially associated with a corresponding DAPI-positive cell. PCNA/GCG or CC3/GCG co-expression was assigned only when the PCNA or CC3 signal could be confidently attributed to the same DAPI-defined GCG-positive cell. Cells with ambiguous staining, overlapping nuclei, fragmented signal that could not be assigned to a single cell, or poor nuclear morphology were excluded from quantification.

### Quantitative RT-PCR

Total RNA was extracted from isolated islets of adult mice, whole pancreatic tissues of neonatal mice, and TC1.6 cells using TRIzol reagent (Invitrogen) according to the manufacturer’s instructions. Complementary DNA (cDNA) synthesis was performed using High-Capacity cDNA Reverse Transcription Kit (Applied Biosystems). Quantitative PCR was performed in triplicate using SYBR Green Master Mix (Abclonal) on a Roto Real-Time PCR System (Qiagen). Gene expression levels were normalized to Gapdh, and relative expression was calculated using the 2-ΔΔCt method [19]. Primer information can be found in supplementary material 1.

### Blood glucose and serum glucagon/insulin assays

Blood glucose was measured immediately after mice were sacrificed and before serum preparation. Briefly, blood was collected by eyeball enucleation immediately after sacrifice. A small aliquot of fresh blood was used for immediate glucose measurement, while the remaining blood was processed for serum collection. Serum glucagon and insulin levels were then quantified using a mouse glucagon ELISA kit (Abclonal) and a mouse insulin ELISA kit (Proteintech), respectively.

### Glucagon secretion assays

For islet secretion assays, isolated islets were pre-incubated in Krebs-Ringer bicarbonate (KRB) buffer with 3 mM glucose for 1 h, followed by incubation in KRB containing either 1 mM or 16 mM glucose. Secreted glucagon in the supernatant was measured by ELISA. Glucagon secretion at 1 mM or 16 mM glucose was expressed relative to the 3 mM glucose baseline obtained during the pre-incubation step. For TC1.6 secretion assays, cells were washed and pre-incubated in KRB buffer containing 3 mM glucose for 1 h, followed by incubation in KRB with either 1 mM or 16 mM glucose using the same protocol as for isolated islets. Glucagon released into the supernatant was quantified by ELISA and expressed relative to the 3 mM glucose baseline.

### TC1.6 cell culture and treatments

TC1.6 cells were maintained in Dulbecco’s Modified Eagle Medium (DMEM) supplemented with 10% fetal bovine serum (FBS) and 1% penicillin-streptomycin at 37 °C in a humidified atmosphere containing 5% CO₂.

For genetic knockout of BASIC, TC1.6 cells were transfected with a PX458-GFP plasmid (Addgene plasmid #48138) encoding CRISPR-Cas9 and a sgRNA targeting the BASIC gene, using Lipofectamine 2000 (Thermo Fisher Scientific) according to the manufacturer’s protocol. GFP-positive cells were sorted by fluorescence-activated cell sorting (FACS) and expanded. Successful knockout was confirmed by genomic DNA sequencing before proceeding to downstream experiments.

### Cell proliferation assays

Cell proliferation was assessed using the Cell Counting Kit-8 (CCK8; MedChemExpress) according to the manufacturer’s instructions. Absorbance at 450 nm was measured using a microplate reader after incubation with CCK8 reagent for 2 h.

### Statistical analysis

All experiments were performed with at least three independent biological replicates. Data are presented as mean ± standard error of the mean (SEM). Statistical analysis and graph generation were performed using GraphPad Prism software (version9.1, GraphPad Software). Comparisons between two groups were made using unpaired two-tailed Student’s t-tests, unless otherwise specified. A p-value < 0.05 was considered statistically significant.

## Supplementary Information

Below is the link to the electronic supplementary material.

Supplementary Material 1 (DOCX 16.2 KB)

## Data Availability

All data supporting the findings of this study are available within the paper.
