# Supplementary material for: The bile acid-sensive ion channel (BASIC) is expressed in pancreatic α-cells and involved in glucagon secretion
Source: Pflugers Arch. 2026 Jul 29;478(8):70. doi: 10.1007/s00424-026-03199-4 (PMC13415342; doi:10.1007/s00424-026-03199-4)
Supplement: Supplementary file 1 — Supplementary Material 1 (DOCX 16.2 KB) [file 424_2026_3199_MOESM1_ESM.docx]

Table 1. RT-PCR primer for genotyping.

| Primer name | Sequence |
| --- | --- |
| mBASIC forward | AGCTGCTCCTCGACGTCCATCA |
| mBASIC reverse | ATGCCGGGCTTTGCACTCCTTC |

Table 2. qPCR primer.

| Primer name | Sequence |
| --- | --- |
| mARX forward | CTGCTGAAGCGCAAACAGAGGC |
| mARX reverse | CTCTGTCAGGTCCAGCCTCATG |
| mPAX forward | AGCTCAGATGCGACTTCAGC |
| mPAX reverse | ATGGGTCCTCTCAAACTCTTTCT |
| mPou3f4 forward | GTATGGCAACGTGTTCTCGCAG |
| mPou3f4 reverse | TCCTGTGGATGAATCAGCCTCC |
| mIRX forward | GAGGAGAGTTCAGACAAGGCAC |
| mIRX reverse | AGCCTGATTCGCACAAGGCATC |
| mGAPDH forward | CATCACTGCCACCCAGAAGACTG |
| mGAPDH reverse | ATGCCAGTGAGCTTCCCGTTCAG |
| mBeta-actin forward | CATTGCTGACAGGATGCAGAAGG |
| mBeta-actin reverse | TGCTGGAAGGTGGACAGTGAGG |

Table 3. qPCR Taqman probe.

| Probe name | Assay ID | Company |
| --- | --- | --- |
| GAPDH | Mm99999915_g1 | Thermo Fisher Scientific |
| GCG | Mm00801714_m1 | Thermo Fisher Scientific |
| BASIC | Mm00517541_m1 | Thermo Fisher Scientific |
